# Enhancing intrusion detection in wireless sensor networks using a Tabu search based optimized random forest

**DOI:** 10.1038/s41598-025-03498-3

**Published:** 2025-05-28

**Authors:** Vivek Kumar Pandey, Shiv Prakash, Tarun Kumar Gupta, Priyanshu Sinha, Tiansheng Yang, Rajkumar Singh Rathore, Lu Wang, Sabeen Tahir, Sheikh Tahir Bakhsh

**Affiliations:** 1 Department of Electronics and Communication, University of Allahabad, Prayagraj, India; 2 Department of Computer Science, Miranda House, University of Delhi, Delhi, India; 3 University of South Wales, Llantwit Rd, Pontypridd, CF37 1DL UK; 4 Department of Computer Science, Cardiff School of Technologies, Cardiff Metropolitan University, Llandaff Campus, Western Avenue, Cardiff, CF5 2QS UK; 5 Xi’an Jiaotong-Liverpool University, Suzhou, China

**Keywords:** Wireless sensor networks (WSNs), Intrusion detection, WSN-DS, Tabu search, Random forest, Optimization

## Abstract

Intrusion detection in Wireless Sensor Networks (WSNs) is an emerging area of research, given their extensive use in sensitive fields like military surveillance, healthcare, environmental monitoring, and smart cities. However, WSNs face several security challenges due to their limited computational capabilities and energy constraints. Their deployment in open, unattended environments makes them especially vulnerable to threats like eavesdropping, interference, and jamming. To address this problem, Random Forest (RF) is a popular machine learning model. The RF model can be tweaked because of its multiple hyperparameters. Tuning these parameters manually is tedious, as the combinations will be exponential. This work presents an enhanced intrusion detection approach by integrating Tabu Search (TS) optimization with a RF classifier. As a result, TS will help RF automatically search optimal hyperparameters and improve the generalization ability. This work integrates the pros of TS with RF. The model was tested on three different datasets, i.e., (a) the WSN-DS dataset, (b) CICIDS 2017, and (c) the CIC-IoT 2023 dataset, which shows better results on different metrics like precision, recall, F1-score, Cohen’s Kappa, and ROC AUC. Detection of Blackhole and Gray Hole attacks also improved, demonstrating the effectiveness of combining metaheuristic optimization with ensemble learning for stronger WSN security.

## Introduction

**Fig. 1:**
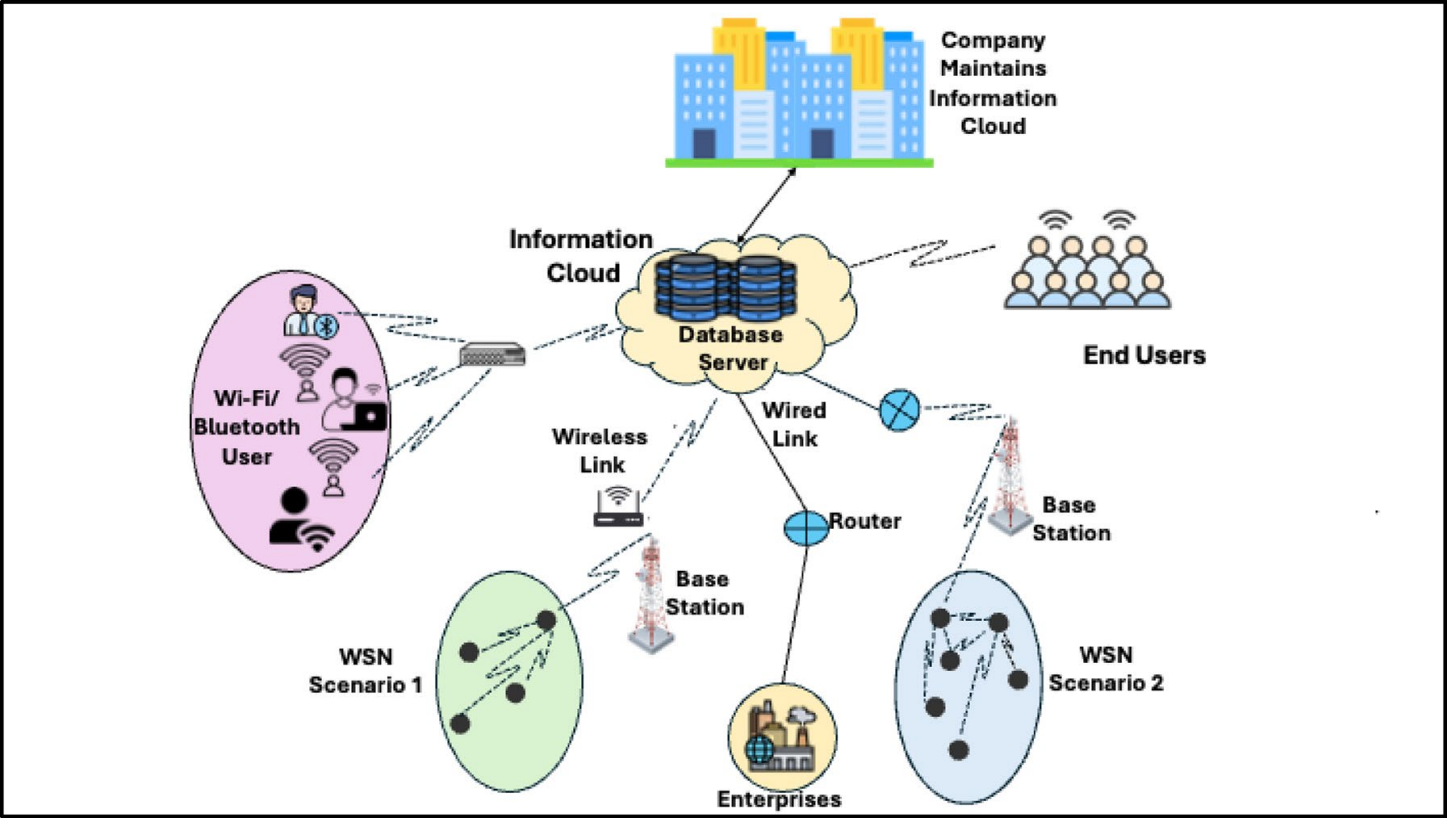
WSN with its applications.

**Fig. 2:**
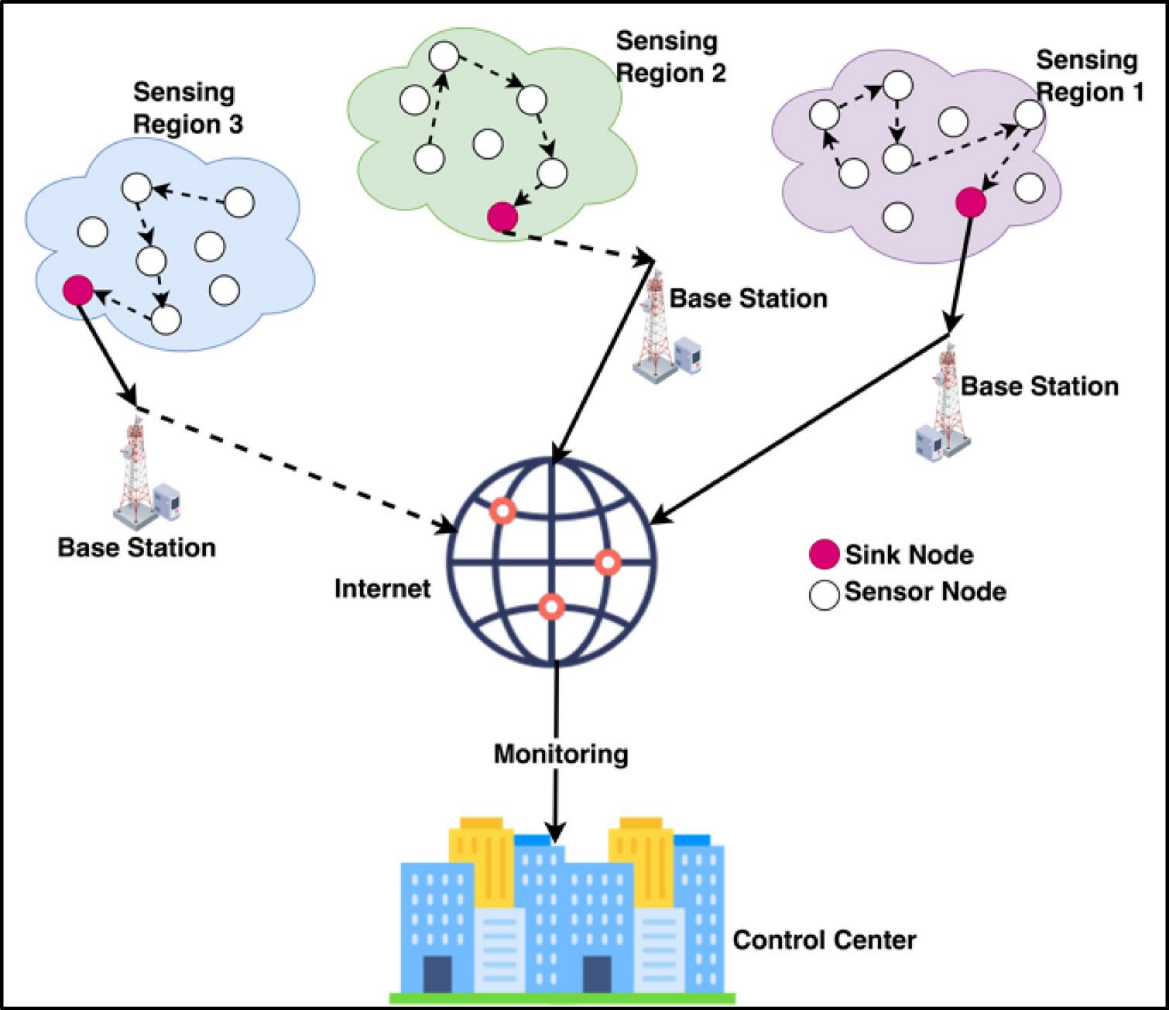
The WSN architecture.

Wireless Sensor Networks (WSNs) are becoming very important in many applications, including environmental monitoring, industrial automation, and smart cities^1,2^ as shown in Fig. 1. It can be defined as a network composed of sparsely distributed, in some cases remote, wireless sensors that cooperate to potentially monitor physical or environmental conditions, such as temperature, humidity, sound, vibration, pressure, motion, or any other environmental or physical parameter^3^. In WSN, several sensor nodes collect the data from the environment and forward it to the gateway or base station. The base station analyzes and processes the collected data and computes valuable information. After that, the base station transmits this data to the control center. The administrator at the control center uses this information for further action. The architecture of WSN is shown in Fig. 2. Moreover, like other networks, it also suffers from the open nature of wireless communication and sensor nodes’ severe resource constraints, making them particularly vulnerable to various security threats^4,5^. Intrusion detection systems are acquiring significant importance within such networks. Recent works on enhanced accuracy and efficiency of intrusion detection in WSNs leveraged machine learning techniques based on ensemble methods such as RF^6^. Distributed wireless sensors monitor the environmental and physical parameters in WSN. Thus, they resist various types of attacks, such as Blackhole attacks, Flooding attacks, Gray-hole attacks, and TDMA attacks^7^. While significant advances have been made in WSN security, most existing approaches to intrusion detection have substantial challenges in reaching higher accuracy and exerting acceptable computational efficiency, especially in resource-constrained environments^8^.

Most existing solutions cannot adapt dynamically to changing attacks and network conditions, thus leading to higher false positives and missed detections^9^. Nonetheless, machine learning model optimization in intrusion detection for WSN has been left under-explored, with most studies focusing on the choice of algorithm rather than on tuning the parameters used^10^.

### Motivation and challenges

As the nature of WSNs is open, they are vulnerable to threats such as eavesdropping, spoofing, and jamming^11^. These vulnerabilities are further exacerbated by sensor nodes’ limited computational power and energy constraints, which restrict the implementation of complex security protocols^12^. Another significant challenge in securing WSNs is achieving high intrusion detection accuracy without compromising computational efficiency, as resource-intensive methods can quickly drain node energy and degrade network performance^13^. Also, many existing security mechanisms are static and struggle to adapt to the dynamic nature of modern cyberattacks and fluctuating network conditions^14^. This lack of adaptability increased undetected threats, undermining the reliability of the network’s defense system.

The novelty of this work is to fill these gaps by proposing a novel approach in which the advantages of TS are combined with the RF algorithm and tested over three datasets: WSN-DS, CICIDS 2017, and CIC-IoT 2023.The main goals of this work are as follows:

- To optimize intrusion detection in WSNs for high accuracy and efficiency using an optimized RF model.
- To validate the utility of TS in fine-tuning RF parameters to improve performance.
- The proposed approach is evaluated on the extensive WSN-DS, CICIDS 2017, and CIC-IoT 2023 datasets, giving an idea about its feasibility over various types of attacks.

This research work contributes to the field of WSN security in the following areas:

- Proposing a Tabu Search-optimized RF model that outperforms standard implementations, achieving superior accuracy, precision, recall, ROC AUC, Cohen’s kappa, and F1-score values.
- A detailed study of intrusion detection behaviors and performance across all scenarios related to types of attacks in WSN, particularly Blackhole, Flooding, Grayhole, and TDMA attacks.
- Providing some insights about the impact of hyperparameter optimization on intrusion detection performance in resource-constrained environments.

This work expands on recent advancements toward WSN security and optimization scenarios in machine learning by accommodating more robust and adaptive intrusion detection systems.

The organization of the paper is as follows. After the introduction in Section "Introduction", the literature review is elaborated in Section "Proposed methodology".The proposed model and data analysis are described in Sect. 3.The mathematical modeling is presented in Section "Conclusion and future scope".Further, Sect. 5 provides an experimental evaluation and comprehensive analysis of the results. Section 6 provides comparison with state-of-the-art techniques. Finally, Sect. 7 concludes the manuscript and highlights its scope for the future.

## Literature review

According to the literature, many researchers have explored machine learning models that aim to design optimal intrusion detection systems to achieve high intrusion detection accuracy and reduce vulnerability issues. Still, the task is very complex and challenging. This section presents a comparative analysis of similar algorithms addressing this problem. All key advancements reviewed in WSN security from 2018 to 2025, along with machine learning applications in intrusion detection, with optimizations followed for classification algorithms.

In the literature, SVM-based IDS has achieved 90.8% accuracy using this model^15^. Among the energy efficiency methods, the Firefly algorithm remained an interesting one that achieved a packet delivery ratio of 91.52% and extended the network lifespan by as much as 89.95%^16^. With the help of blockchain and federated learning, 100% accuracy for binary classification and 99.95% for multiclass has been achieved to classify malicious nodes in IoT^17^. However, techniques like RF, gradient boosting, and SVM have also been elaborated for security-enhanced and localization-based improvements^18^. In addition, proper data transmission and energy consumption were guaranteed with the help of trust evaluation and cascading encryption^19^. Different DoS attacks in IoT networks are done using the application of XGBoost for feature selection compared with Naive Bayes and Recurrent Neural Networks^20^. RNN, in an attempt to take snapshots of time patterns, showed outstanding performance at 99.7 percent compared to NB, which reached a reasonable accuracy of 97.9 percent; therefore, RNN could be very effective in detection in IoT environments^21^.

Furthermore, multiple intrusion detection strategies for IoT ecosystems were evaluated, exploiting quantitative metrics comprising accuracy, precision, recall, F1-score, and MCC^22,23^. The performance results of the machine learning-based IDS models mostly depicted high accuracy values of over 99%, especially when ensemble techniques such as RF and XGBoost were adopted in the models^24^. Instead, feature engineering and transfer learning improvements were emphasized and left as future optimization work^25^. Another model has been proposed that achieved high performance in intrusion detection. Results showed 99.7% accuracy, 99.8% precision, and 97.8% recall on the NSL-KDD dataset^26^. The same high outcomes were seen on other datasets, such as UNSW-NB15 and CICIDS 2017, with the model’s efficacy in improving detection accuracy through feature reduction and data balancing techniques^10,27^.

The WSN intrusion detection model is enhanced using an optimization technique, PL-AOA, for the kNN algorithm. It achieved 99% detection accuracy against DoS attacks^28^. Using traditional kNN, this design enhanced the detection accuracy by 10%.It realized a balance between lightweight computation and high intrusion performance^14,29^. Another study proposes a novel technique for IoT-WSN intrusion detection using the BCOA-MLID technique, which uses a binary chimp optimization algorithm and machine learning. This approach outperformed the existing methods, like XGBoost and KNN-AOA, since it achieved a maximum accuracy of 99.36%.The results indicated that BCOA-MLID exhibited better classification metrics than sensitivity, specificity, F-score, and AUC. Its computation time was meager, approximately 7.26 s, which made it suitable for IoT-WSN with highly constrained resources^28,30,31^. Moreover, theFA-ML-based intrusion detection technique in WSN-IoT integrates the firefly algorithm and machine learning^32–35^. The study in^36^ further proved that the developed ensemble model in experiments on intrusion detection outperformed the single classifiers, and the accuracy rate reached up to 98.8% by using techniques of KNN and boosting. Precision and recall rates had reached up to 97%.In boosting and stacking ensemble methods, the F1 scores reached 96% and 97%, respectively. This model can be highly efficient in detecting cyberattacks in IoT environments. The ensemble-based machine learning model using RF proposed in^34,37^ surpasses the existing intrusion detection methods^38–40^ by achieving over 99% accuracy and a false positive rate. Also, it mitigated the previously prevalent drawbacks in these systems and provided a reliable framework for intrusion detection across various public datasets. Many researchers have tried to optimize the RF algorithm, but due to certain limitations, the best optimal solution cannot be obtained^41–43^. With an exhaustive literature review, it can be concluded that there exist so many critical research gaps, such as (a) much research focuses on selecting different algorithms, the critical aspect of tuning these algorithms for the specific context of WSN security to maximize performance requires (b) No Exploring and optimizing hybrid intrusion detection modelsand (c) Additionally, feature engineering plays a vital role in improving the effectiveness of Intrusion Detection Systems (IDS), this area needs more consideration.

The contribution of this work is to use a metaheuristic Tabu-Search approach^44^ for optimizing the performance of the RF method in intrusion detection by WSN. It results in better performance than the basic RF approach^45^. This proposed technique is tested in three benchmark datasets: the WSN-DS dataset^46,47^, the CICIDS 2017^48,49^, and the CIC IoT 2023 dataset^50,51^. A summary of reviewed work has been presented in Table 1.

**Table 1:** Literature review summary of ML-based intrusion detection systems in WSN/IoT.

Reference	Research gap	Techniques used	Research findings	Limitations
^15^	Limited accuracy in traditional IDS approaches	SVM-based IDS	Achieved 90.8% accuracy	High computational complexity; limited feature selection
^16^	Energy consumption issues in WSN security	Firefly algorithm for energy efficiency	91.52% packet delivery ratio; extended network lifespan by	Trade-off between security and energy conservation; not ideal for dense networks
^17^	Centralized architecture vulnerabilities	Blockchain and federated learning	100% accuracy for binary classification; 99.95% for multiclass classification	High resource requirements; latency issues in large-scale IoT deployment
^18^	Insufficient feature engineering	RF, gradient boosting, and SVM	Enhanced security and localization improvements	Less effective in detecting zero-day attacks; dataset imbalance issues
^19^	Data integrity during transmission	Trust evaluation and cascading encryption	Guaranteed proper data transmission and energy	Key management overhead, increased latency in data transmission
^20^	DoS attack detection accuracy	XGBoost for feature selection, compared with NB and RNN	Superior performance of XGBoost in feature selection	High memory requirements; performance degradation with limited training data
^21^	Temporal pattern recognition in attacks	RNN compared to Naive Bayes	RNN: 99.7% accuracy; NB: 97.9% accuracy	RNN training complexity: resource intensive for resource-constrained IoT
^22,23^	Lack of standardized evaluation metrics	Multiple IDS strategies with quantitative metrics	A comprehensive evaluation framework was established	Inconsistent results across different datasets; metric selection bias
^24^	Single classifier limitations	Ensemble techniques (RF and XGBoost)	> 99% accuracy with ensemble methods	Model complexity; difficult to implement in resource-constrained environments
^25^	Limited feature engineering	Feature engineering and transfer learning	Improved model generalization	Lack of optimized feature selection; transfer learning limitations across domains
^26^	Performance on diverse datasets	ML-based IDS with feature reduction	99.7% accuracy, 99.8% precision, 97.8% recall on NSL-KDD	Dataset-specific optimizations that may not generalize well
^27^	Data imbalance issues	Feature reduction and data balancing	High performance on UNSW-NB15 and CICIDS 2017	Complex preprocessing requirements; limited real-time detection capability
^28^	kNN optimization for WSN	PL-AOA optimization for kNN	99% detection accuracy against DoS attacks; 10% improvement over traditional kNN	Limited to specific attack types; performance varies with network topology
^29^	Balancing computation and accuracy	Lightweight computation optimization	Balance between resource usage and detection performance	Trade-off between lightweight nature and detection capability
^34,35^	Integration challenges in WSN-IoT	FA-ML (Firefly Algorithm with ML)	Improved detection rates with optimized energy consumption	Algorithm convergence issues in dynamic network conditions
^41^	RF optimization limitations	RF parameter tuning approaches	Improved RF performance, but not optimal	Optimization method limitations: local optima problems
^42^	Feature relevance in RF models	Feature importance analysis with RF	Better understanding of attack signatures	Limited dynamic adaptation to emerging threats
^43^	Hyperparameter tuning	Grid search and randomized search for RF optimization	Improved model performance	Computationally expensive optimization; not ideal for resource-constrained WSNs
^44^	Metaheuristic optimization for IDS	Tabu-Search approach	Enhanced optimization performance	Search space limitations; potential convergence to local optima
^45^	RF baseline performance	Basic RF implementation	Established performance baseline	Non-optimized hyperparameters; overfitting on certain datasets
^46,47^	Dataset benchmarking	WSN-DS dataset evaluation	Standard dataset for WSN security evaluation	Limited attack diversity; potential dataset bias
^48,49^	Real-world attack scenarios	CICIDS 2017 dataset implementation	Realistic network traffic patterns	Dataset aging; some attack vectors becoming outdated
^50,51^	IoT-specific attack patterns	CIC IoT 2023 dataset evaluation	Latest IoT attack patterns and signatures	Latest IoT attack patterns and signatures

## Proposed methodology

In machine learning, models must deal with numerous parameters and dozens of hyperparameters. The combinations of these parameters and hyperparameters became exponential, so selecting the correct combination became nearly impossible if anyone followed a manual design approach. Since models like RFs consist of about 19 hyperparameters, designing an ideal model manually with the correct number of hyperparameters will be time-consuming and tedious. This section introduces the TS algorithm and the RF technique, particularly emphasizing how TS could improve hyperparameter optimization. Firstly, this paper discusses the TS algorithm and its basic properties, as well as the general process of its work. After that, the section proceeds to a more general description of the RF method and its properties. Finally, the new proposed method will be described in greater detail, how TS is combined with RF, with the goal of optimization.

### Tabu-search (TS)

Tabu Search^10^ is a metaheuristic optimization technique adopted to solve combinatorial optimization problems. The TS algorithm iteratively explores the solution space in the hope that, at each step, a better candidate solution is found while avoiding getting trapped in local optima. A unique feature of the TS algorithm is the memory structure known as the “tabu list” so as not to backtrack to any previously explored solution. TS incrementally explores the solution space using move operators to generate new potential solutions and evaluate outcomes. The algorithm tracks the solutions already studied in the search process along with the tabu list.

### Random forest

RF^14,52^ is highly popular in machine learning for classification and regression problems since it gives the ensemble method: building a “forest” of decision trees from subsets of data and features chosen randomly, averaged to make a prediction. This random selection process is based on the principle that decision trees can produce more accurate predictions when they use different subsets of data and features than when relying upon a single decision tree. Reducing overfitting and enhancing generalization capabilities are the benefits of learning in RF. The main benefits of RF in the proposed methodology are as follows: (a) ease of use, (b) minimal preprocessing requirements, and (c) ability to handle both categorical and continuous input features.

### Optimization methodology

**Fig. 3:**
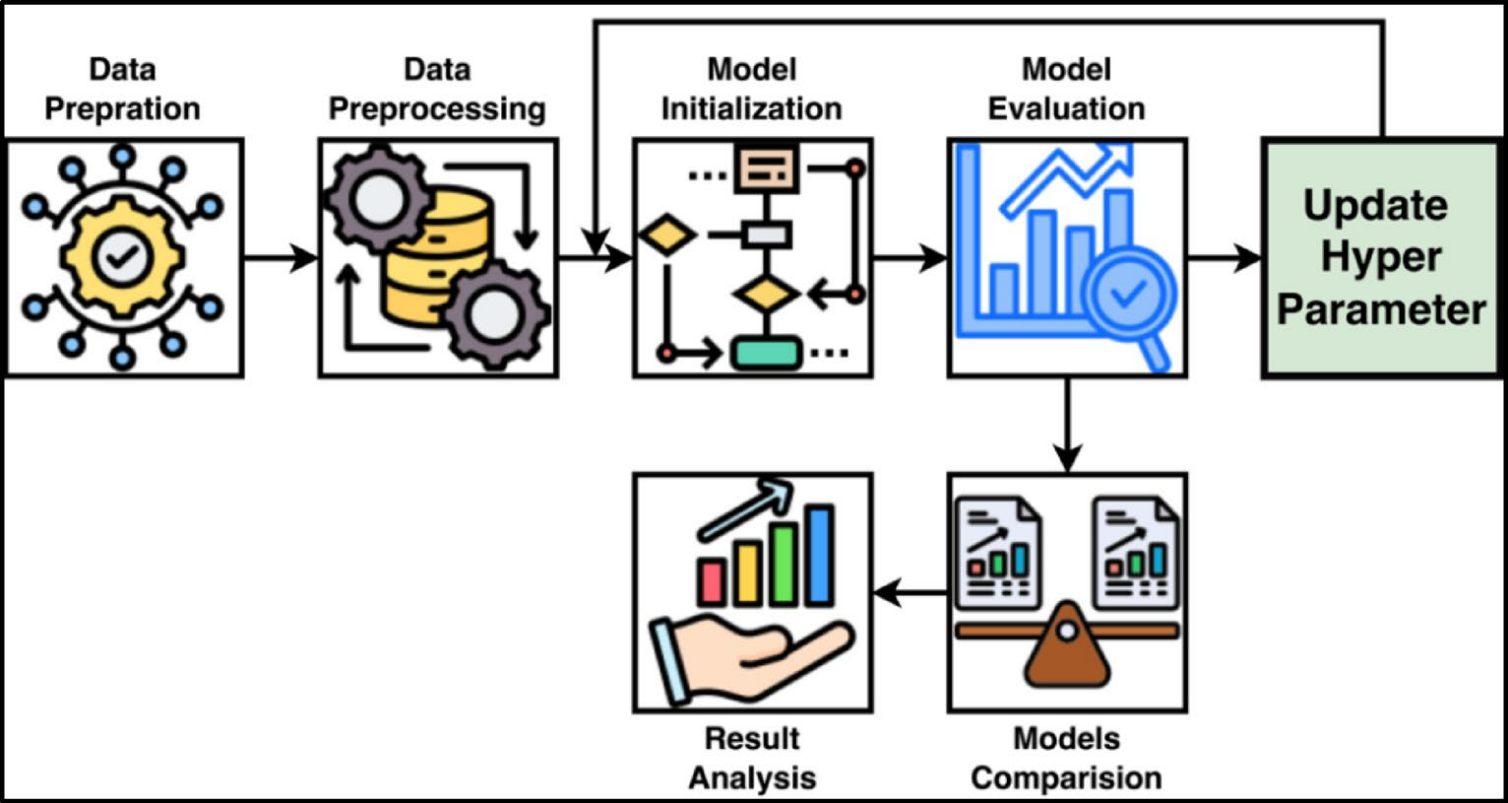
Flowchart of proposed methodology.

A novel approach based on tabu search optimization^53^ has been introduced for optimizing RF models. The main idea is to combine two powerful concepts:RF’s Parameter Space and Tabu Search as an Efficient Navigator. RFs have many parameters, known as hyperparameters (as many as 19), that are hard to tune because they create a gigantic and complex space of possible combinations. Tabu Search helps intelligently navigate this space without becoming stuck in local optima. It remembers the settings it has previously attempted (in a “tabu list”) and does not repeat them, which is time-saving and helps find better solutions. This makes it a valuable technique for discovering the optimal settings for a RF model, particularly when straightforward techniques such as trial-and-error or grid search are ineffective. The flowchart in Fig. 3 illustrates this optimization method in a step-by-step way.

#### Data preparation

The WSN-DS dataset is comprised of 374,661 entries over 19 columns. Features are related to wireless sensor network behavior with “Attack type” as the target variable.

#### Data preprocessing

The dataset was read into pandas; the column “Attack type” was separated from the rest of the feature set. Further, the data were split into training and testing sets with features (X) and target variables (y) separated over an 80–20 split.

#### Initial model development

A RF classifier is implemented with initial hyperparametersn_estimators = 100, max_depth = 10, and was trained on the training data.

#### Model evaluation

The model was assessed using the metrics mentioned: accuracy, precision, recall, F1 Score, Cohen’s Kappa, and ROC AUC. A confusion matrix and ROC curve were also plotted for visualization.

#### Hyperparameter optimization

A Tabu Search algorithm was defined and implemented forhyperparameter tuning, where n_estimators and max_depth were experimented with; the best fitting model was selected by maximizing precision.

#### Optimized model development

The optimized parameters-hyperparameters were used to develop a new RF Classifier, which was later trained on training data.

#### Comparative analysis

The same set of evaluation metrics was used for the original and optimized models for the comparison. Confusion matrices and ROC curves were drawn to better visualize the correctlyclassified instances and class differentiation. The classification report offers a detailed overview of the performance by class in terms of precision, recall, F1 score, and support.

#### Results interpretation

The performance of the original and optimized models was compared to determine whether the optimization process had an effect. It could be seen that improvements were made across several metrics, so it is safe to claim the optimized model performs better than the original. Perhaps most importantly, the optimized model handled the minority classes much better, which is often a problem when addressing imbalance in a dataset.

## Mathematical modeling of RF optimization using Tabu-search

### Objective function

The optimization model mainly aims to maximize classification performance as several key performance metrics are integrated, each assigned a weighted importance. The model maximizes the function given in Eq. 1 below:

1 $$F\left(\theta \right)= {w}_{1}A\left(\theta \right)+ {w}_{2}P\left(\theta \right)+{w}_{3}R\left(\theta \right)+{w}_{4}{F}_{1}\left(\theta \right)+{w}_{5}K\left(\theta \right)+{w}_{6}ROC\left(\theta \right)$$

where $\theta =\left(n,d\right)\epsilon S$ represents the solution vector, with n as the number of estimators and d as the maximum depth of the model. Each element in the function corresponds to a performance metric:$A\left(\theta \right)$ is accuracy, calculated as follows:$(TP+TN)/(TP+TN+FP+FN)$; $P\left(\theta \right)$ is precision described as $TP/TP+FP$; $R\left(\theta \right)$ is the recall can be represented as $TP/TP+FN;{F}_{1}\left(\theta \right)$ is the F1 score, balancing both precision and recall as $2(P*R)/P+R$; $K\left(\theta \right)$ is Cohen’s Kappa, which measures the agreement between prediction and actual outcomes on anything other than a chance basis, as $({P}_{0}-{P}_{e})/(1-{P}_{e})$; and $ROC\left(\theta \right)$ is the area under the receiver operating characteristic, or AUC, understood as the integral of the true positive rate to the false positive rate. Letting the weights be ${w}_{1}, {w}_{2}, {w}_{3}{, {w}_{4, }w}_{5}, {w}_{6}$ the model allows for varying degrees of aspect importance to classification performance through the solution vector $\theta .$

### Constraints

The optimization model is constrained over the solution vector,$\theta =\left(n,d\right)$ with positive integers: the number of estimators n and the maximum depth d, that is, $n, d\in {\mathbb{Z}}^{+}.$ Further, the number of estimators will lie in between lower and upper bounds ${n}_{min}\le n\le {n}_{max}$ and the depth will then also lie in between the lower and upper bounds of ${d}_{min}\le d\le {d}_{max}$. Due to these bounding functions, the optimization process will remain within feasible and practical limits for the model parameters.

### Search space

The model outlines potential solutions and delineates the search space for optimization. The solution space S is given in Eq. 2 as:

2 $$S=\left\{(n, d)|n, d\in {\mathbb{Z}}^{+}, {n}_{min}\le n\le {n}_{max}, {d}_{min}\le d\le {d}_{max}\right\}$$

It is a set of all pairs $\left(n,d\right)$, such that $n$ and $d$ are positive integers and represent the number of estimators and the maximum depth, respectively. They are subjected to the corresponding bounds. Furthermore, for every solution $\theta$, the neighborhood $N(\theta )$ denotes the set of all solutions thatsatisfy ${\theta }{\prime}\epsilon S$ such that $\Vert {\theta }{\prime}-\theta \Vert \le \Delta ,$ where $\Delta$ represents the extent by which the parameters can be varied during optimization. This then provides the nature of the frame in which optimal solutions could exist and be bounded by the set limits.

### Neighborhood structure

**Fig. 4:**
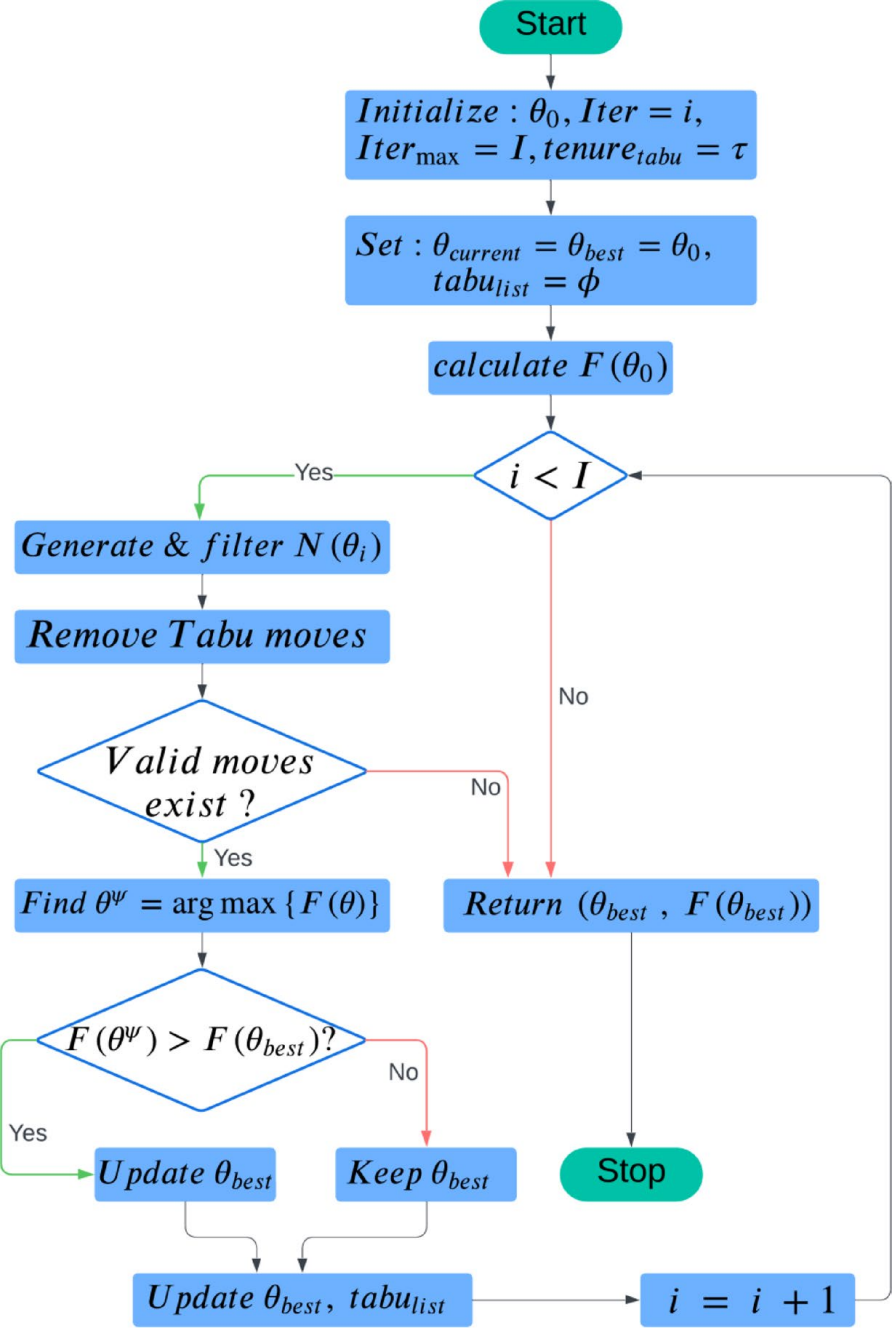
Flow diagram of proposed approach.

The model defines how new candidate solutions are generated by establishing movement patterns within the search space. For a given solution ${\theta }_{i}=({n}_{i}, {d}_{i})$ the neighborhood $N({\theta }_{i})$ consists of solutions that result from small adjustments to the parameters. Specifically, the new candidate solutions are $\left({n}_{i}\pm \Delta n, {d}_{i}\right)$ and $\left({n}_{i}, {d}_{i}\pm \Delta d\right)$, where $\Delta n$ and $\Delta d$ denote allowed deviations in the number of estimators and the maximum depth, respectively. Unless these new solutions fall within the bounds of the solution space $S$, they are valid. This structure guides the search for neighboring solutions during the optimization process. A flow diagram of the proposed approach is shown below in Fig. 4.

**Algorithm 1:**
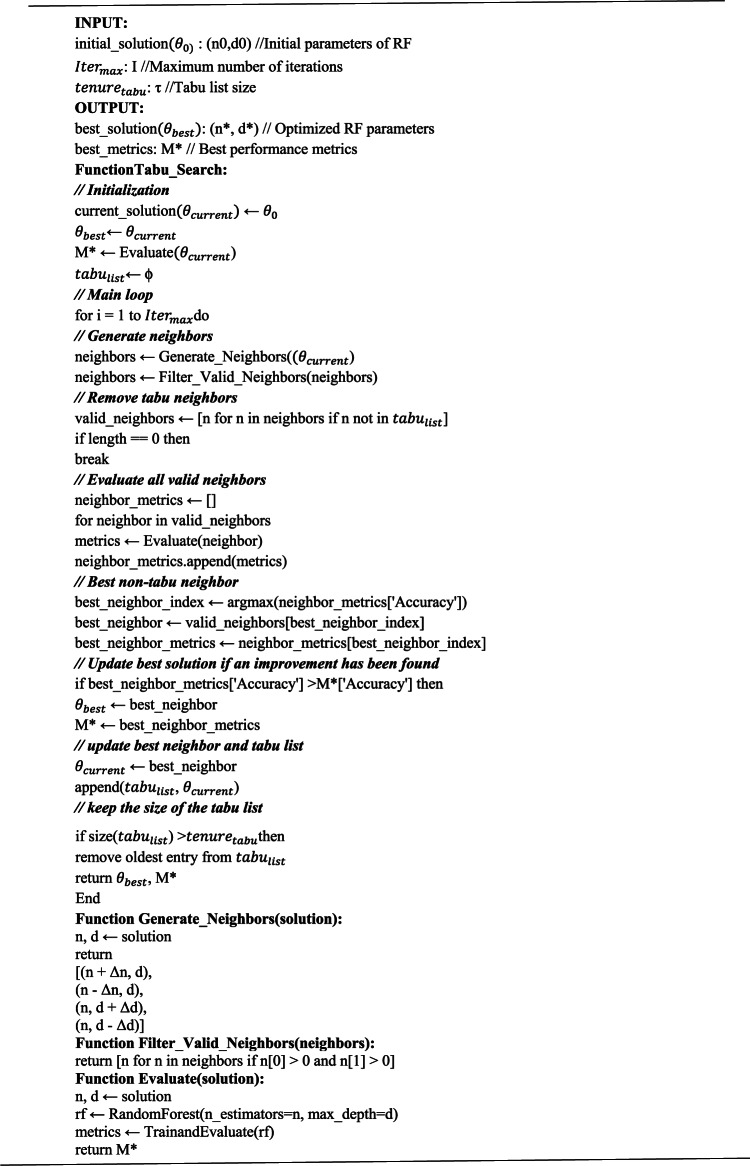
Tabu search for RF optimization

## Results and discussion

### Experimental setup

All the experiments were done in a Python-based environment using Google Colab, which offered the appropriate computational resources for large-scale data of wireless sensor networks. The implementation utilized several specialized libraries of Python: sci-kit-learn version 3.10.12 for machine learning algorithms and metrics; pandas for efficient data manipulation and preprocessing; numpy for numerical computations and array operations; and both matplotlib and seaborn for advanced data visualization. Google Colab’s GPU runtime was applied to speed up the model training process. The local system included a computer running Windows 11 OS. It had an Intel(R) Core(TM) i5-10210U CPU @ 1.60 GHz with four cores and eight logical processors, 4 GB of installed physical memory (RAM), and 500 GB of SSD storage, which provided appropriate resources to start working with data and commence the development of code.

### Dataset characteristics

The proposed approach was tested over three different datasets in further subsections. As the problem is complex, it is required to test the model on multiple datasets to check its generalization and accuracy capabilities.

#### Wireless sensor network dataset (WSN-DS)

The WSN-DShas 374,661 instances, each characterized by 19 distinct features. These features includeId and Time, cluster head information like Is_CH and who CH, and distance-related parameters such as Dist_To_CH and dist_CH_To_BS. The dataset captures communication metrics such as ADV_S, ADV_R, JOIN_S, JOIN_R, SCH_S, and SCH_R, data transmission parameters like DATA_S, DATA_R, and Data Sent to BS. Further, the feature includes Expended Energy and assigns each instance a class label for attack classification to emphasize the importance of network security analysis.

#### Canadian institute for cybersecurity intrusion detection system 2017

The CICIDS 2017 is a well-labeled dataset and is a comprehensive and widely used benchmark dataset for evaluating intrusion detection systems using machine learning and deep learning. This data set is released to simulate real-world benign and malicious traffic in a realistic network environment. It includes both benign and attack traffic. The dataset has 2.8 million instances with 80 + features. The dataset covered mainly DoS attacks, Brute force attacks, web attacks, and DDoS attacks.

#### Comprehensive intrusion detection dataset (CIC-IoT)

The CIC-IoT 2023 was explicitly designed for Internet of Things (IoT) environments. It was released by the Canadian Institute for Cybersecurity (CIC) to support research in detecting cyberattacks on IoT networks using machine learning and deep learning techniques. This dataset is in CSV format with more than 6 million records and 80 + features. The dataset includes DDoS, Botnet, Brute force attacks, web attacks, etc.

### Data preprocessing

Data preprocessing is an essential step in guaranteeing data quality and model performance. First, every dataset was examined for any missing values. In the second step, duplicated or irrelevant features were eliminated to reduce dimensionality and increase model effectiveness. After that, Label encoding was employed to convert categorical variables into numerical form. Finally, the dataset was shuffled and split into training and testing, i.e., 80:20 ratio subsets, to ensure unbiased model evaluation.

### Model performance comparison

The model’s performance is tested over three different datasets, showing that the proposed model is an improved version of the default. All the experiments occurred in two phases. In the first phase, RF with the default hyperparameters is implemented, and then in the second phase, TS is integrated into RF (algorithm 1) for optimal hyperparameters. This work compares the performance of two models of RFs. Table 2 gives a complete comparison of their performance metrics with WSN-DS.

**Table 2:** Performance metrics comparison between initial and optimized RF models over WSN-DS.

Metric	Initial model	Optimized model	Improvement
Accuracy	0.9942	0.9967	+ 0.0025
Precision	0.9943	0.9967	+ 0.0024
Recall	0.9942	0.9967	+ 0.0025
F1 Score	0.9942	0.9967	+ 0.0025
Cohen’s Kappa	0.9667	0.9808	+ 0.0141
ROC AUC	0.9974	0.9980	+ 0.0006

Results obtained in Table 2 depict that the optimized model has a considerable accuracy improvement, from 99.42% to 99.67%, although this only represents a gain of 0.25%; the change corresponds to approximately 187 more correct classifications of 74,933 test samples-a fair amount that improves the effectiveness of the model toward protection against false alarms or missed detections in the context of network security. Precision, recall, and F1 scores improved by about 0.25%.It shows that an optimization enhanced the model’s precision and recall without compromising performance. Cohen’s Kappa score had improved the most with an increase from 0.9667 to 0.9808, meaning that the optimized model has a more robust agreement between the predicted and observed categorizations considering chance, thus making it more potent on multiclass classification tasks. The ROC AUC also increased from 0.9974 to 0.9980, improving the distinction between classes, especially for less common attack types. Table 3 presents the detailed classification reports for the initial and optimized CICIDS2017 and CIC-IoT-2023 models. It depicts that the proposed optimized model shows better in-case performance metrics for CICIDS2017 and CIC-IoT-2023.

**Table 3:** The results for the initial and optimized CICIDS2017 and CIC-IoT-2023 models.

	CICIDSS2017		CIC-IoT-2023	
Metric	Initial RF	Optimized RF	Initial RF	Optimized RF
Accuracy	0.99	0.99	0.97	0.99
Precision	0.99	0.99	0.97	0.98
Recall	0.99	0.99	0.97	0.99
F1 Score	0.99	0.99	0.96	0.98
Cohen’s Kappa	0.99	0.99	0.96	0.98
ROC AUC	0.99	0.99	0.99	0.99

### Class-wise performance analysis

Tables 4 and 5 present the detailed classification reports for the initial and optimized models.

**Table 4:** Classification report of the initial RF model over WSN-DS.

Class	Precision	Recall	F1score	Support
Blackhole	0.97	1.00	0.98	2043
Flooding	0.90	1.00	0.95	631
Grayhole	0.97	0.94	0.95	2985
Normal	1.00	1.00	1.00	67,965
TDMA	1.00	0.93	0.96	1309
Accuracy	–	–	0.99	74,933
Macro avg	0.97	0.97	0.97	74,933
Weighted avg	0.99	0.99	0.99	74,933

**Table 5:** Classification report of the optimized RF model over WSN-DS.

Class	Precision	Recall	F1-score	Support
Blackhole	0.99	1.00	0.99	2043
Flooding	0.93	0.99	0.96	631
Grayhole	0.98	0.98	0.98	2985
Normal	1.00	1.00	1.00	67,965
TDMA	1.00	0.93	0.96	1309
Accuracy	–	–	1.00	74,933
Macro avg	0.98	0.98	0.98	74,933
Weighted avg	1.00	1.00	1.00	74,933

Classification reports in Tables 4 and 5 show that the RF model performed remarkably well in detecting network attacks and regular traffic in WSN environments with considerable improvements post-optimization. Table 4 shows that the model scored 99% accuracy over 74,933 instances, whereby Normal traffic (67,965 cases; 90.70% dataset) produced perfect precision and recall at 1.00.The recall power of the first model was different for all attack types. Blackhole attacks had 2,043 instances recalled per defect every single time, with a score of 1.00 and a precision of 0.97.Flooding attacks with 631 instances granted perfect recall, but precision was much lower, 0.90.Grayhole attacks had balanced performances at 2,985 cases, as their precision was equal to their recall with scores of 0.97 and 0.94, respectively.

TDMA attacks at 1,309 instances produced perfect precision but relatively low recall at 0.93.After optimization, Table 5 depicts that the model was improved in all metrics, with an ideal accuracy of 1.00 and enhanced performance in detecting the attack. Blackhole attacks have now improved to 0.99 precision while keeping perfect recall. Flooding attacks are now at 0.93 precision with a recall of 0.99.Grayhole attacks had balanced metrics of 0.98 for precision and recall, while TDMA attacks kept the perfect precision and had a recall of 0.93.Macro averages improved from 0.97 to 0.98 while weighted averages increased from 0.99 to 1.00, which indicates the enhanced ability of the model to handle class imbalance and distinguish between the types of attacks, thus making the system of WSN attack detection more robust and more reliable with a minimum number of false positives in all categories.

### Confusion matrix and ROC curve analysis initial RF model

**Fig. 5:**
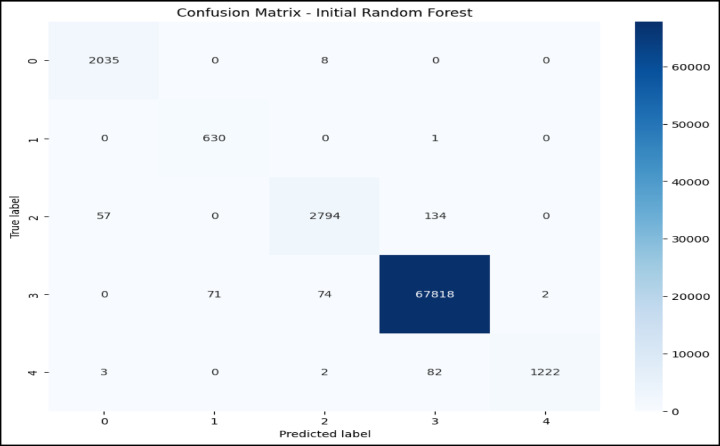
Confusion matrix of initial RF model over WSN-DS.

**Fig. 6:**
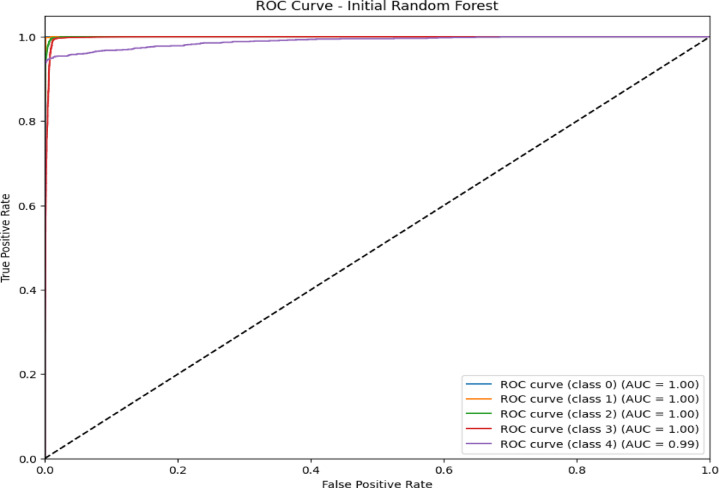
ROC AUC curve of the initial RF model over WSN-DS.

The confusion matrix in Fig. 5 and the ROC curve analysis in Fig. 6 represent the performance of the initial RF model on a multiclass classification problem, which depicts high accuracy for most of the classes. Class 0 (Blackhole attacks) had the correct number of 2,035, with only eight misclassified as class 2 (Grayhole attacks); class 1 (Flooding attacks)was nearly perfect classification with 630 correct predictions and just one being misclassified as class 3(Normal).Class 2 had 2,794 correct classifications, but it showed some confusion, with 57 instances misclassified as class 0 and 134 as class 3.Class 3 had the maximum correct classification,67,818, though 74 cases were misclassified as class 2, and 71 were classified as class 1.Lastly, class 4 (TDMA attacks) was correctly classified 1,222 times, with minor misclassifications into other classes. The ROC curves in Fig. 6 indicate that the model has excellent, near-perfect performance in the classification of Class 0, Class 1, Class 2, and Class 3 with an AUC of 1.00 each, which indicates powerful classification ability between them and the classes. The AUC for Class 4 showed a slightly lower value at 0.99,reflecting high but lesser classification accuracy. Since all the ROC curves lie considerably above the diagonal representing random guessing with an AUC of 0.5, much better-than-chance performance is shown, and all classes are classified with high precision.

### Confusion matrix and ROC curve analysis of optimized RF model

**Fig. 7:**
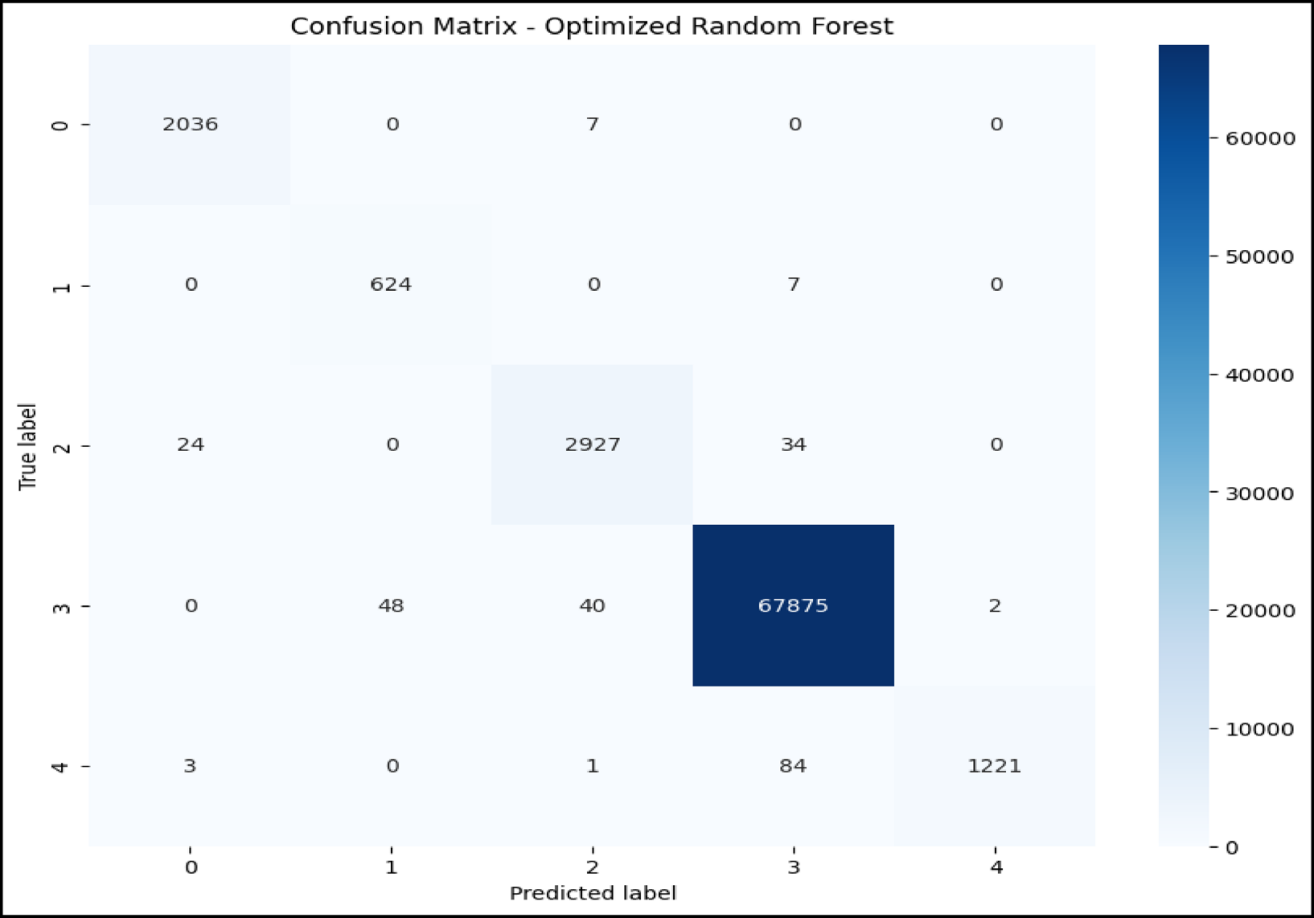
Confusion matrix of optimized RF model over WSN-DS.

**Fig. 8:**
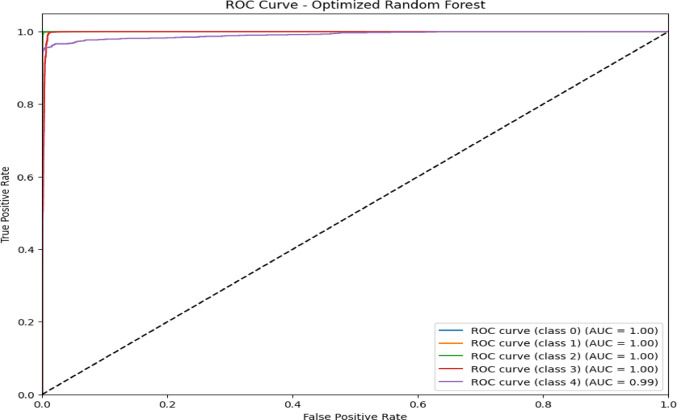
ROC AUC curve of optimized RF Model over WSN-DS.

The confusion matrix in Fig. 7 and the ROC AUC curve in Fig. 8 together explain the performance of the optimized RF model in attack type classification using the WSN-DS, CICIDS 2017, and CIC-IoT 2023datasets: Classes are Blackhole (0), Flooding (1), Grayhole (2), Normal (3), and TDMA (4).The model is excellent at identifying Normal traffic, making 67,875 correct predictions with minimal misclassifications, and also does well in classifying Blackhole attacks with 2,036 correct classifications, although seven are misclassified as Grayhole. Flooding attacks are mostly correctly identified, with 624 correct classifications and 7 misclassifications into TDMA. In comparison, 2,927 correct classifications were made for Grayhole, though 24 fell into the wrong classification of Blackhole, and 34 into the Normal category. TDMA is powerfully and accurately classified (1,221), except for 84 that are misleadingly classified as Normal. Considering this, the ROC AUC curve shows almost perfect classification, which returns an AUC score of 1.00 in classes Blackhole (0), Flooding (1), Grayhole (2), and Normal (3), and with almost no false positives but a high true positive rate. Class TDMA has an AUC of 0.99, which still indicates an excellent classification performance. All classes are well differentiated between the models of type attacks and regular traffic with minimal errors.

**Fig. 9:**
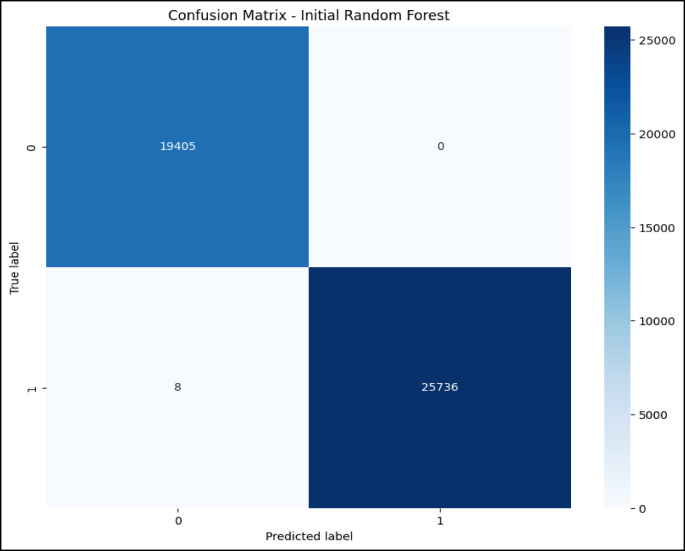
Confusion matrix of initial RF Model over CICIDS 2017.

**Fig. 10:**
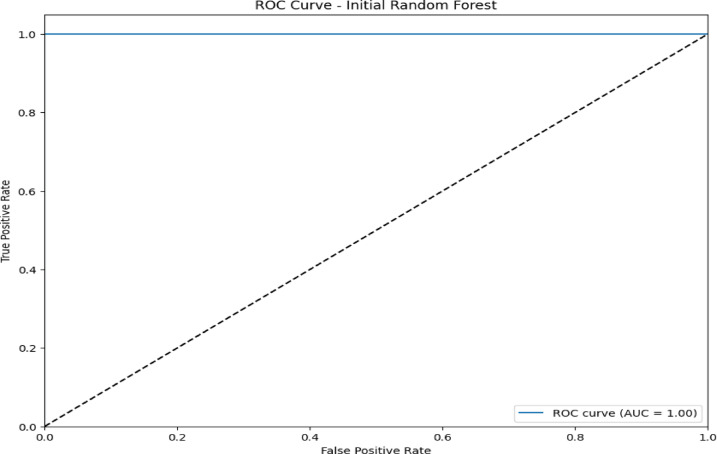
ROC AUC curve of initial RF model over CICIDS 2017.

**Fig. 11:**
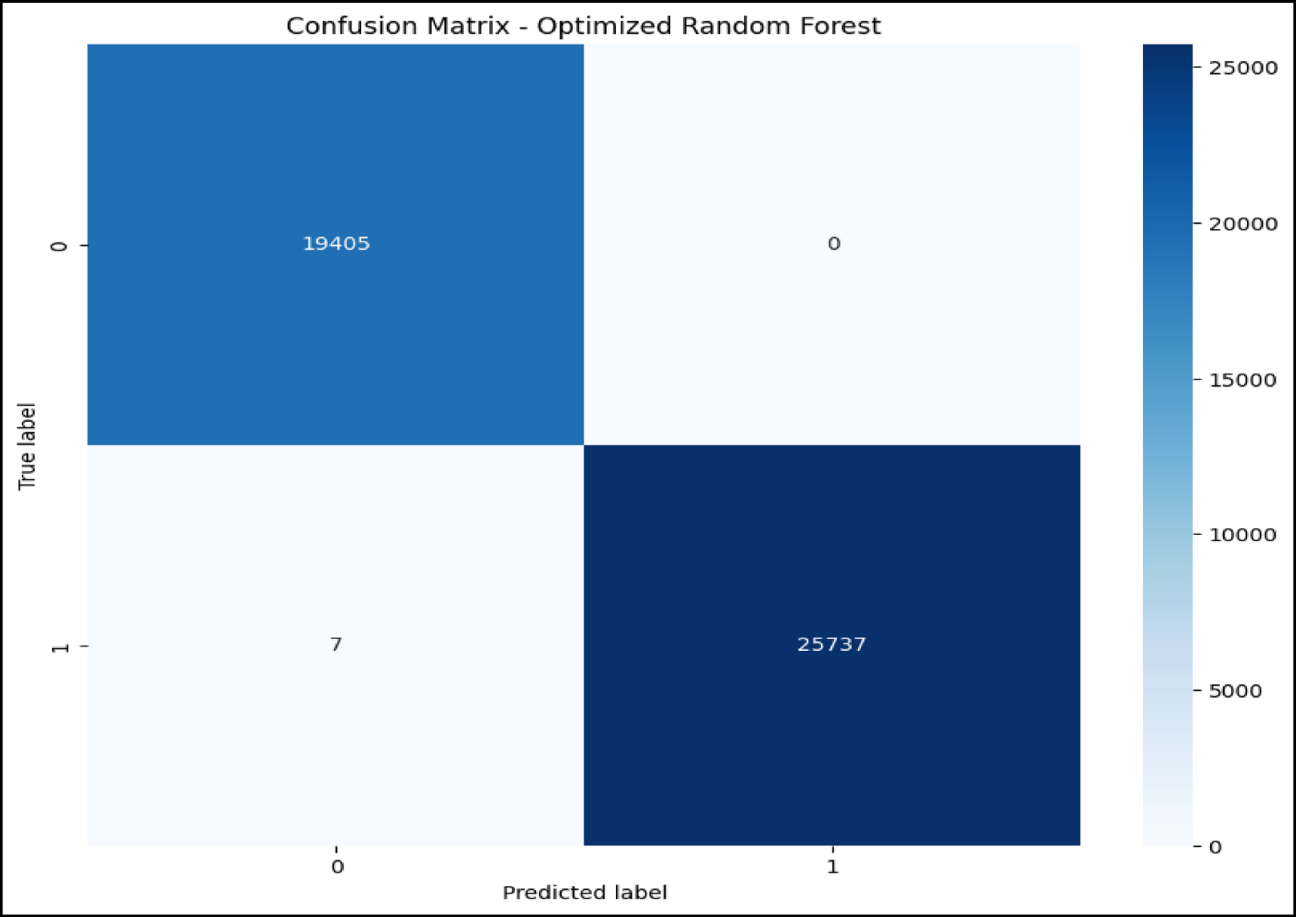
Confusion matrix of optimized RF model over CICIDS 2017.

**Fig. 12:**
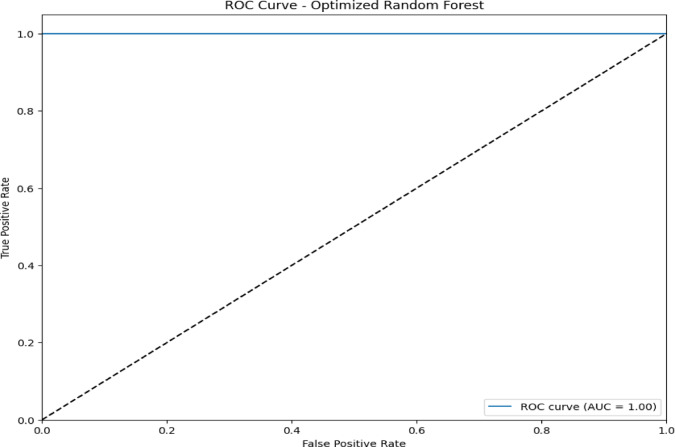
ROC AUC curve of optimized RF model over CICIDS 2017.

Figures 9, 10, 11, 12 illustrate the RF model performance assessment for the CICIDS 2017 dataset with and without optimization. Figure 9 illustrates the original model’s confusion matrix and the accuracy and number of false positives and false negatives. Figure 10 shows the ROC curve AUC metric along with the confusion matrix, showing the ability of the model to truly classify the classes, with 1 indicating perfect performance as described in previous sections. After tuning, Fig. 11 shows the confusion matrix of the optimized model. This likely represents better predictive accuracy and fewer overall errors. Figure 12 shows the corresponding ROC AUC, which shows increases in discrimination after tuning. Figures 9, 10, 11, 12 show a visual snapshot of the RF model performance, before and after tuning.

**Fig. 13:**
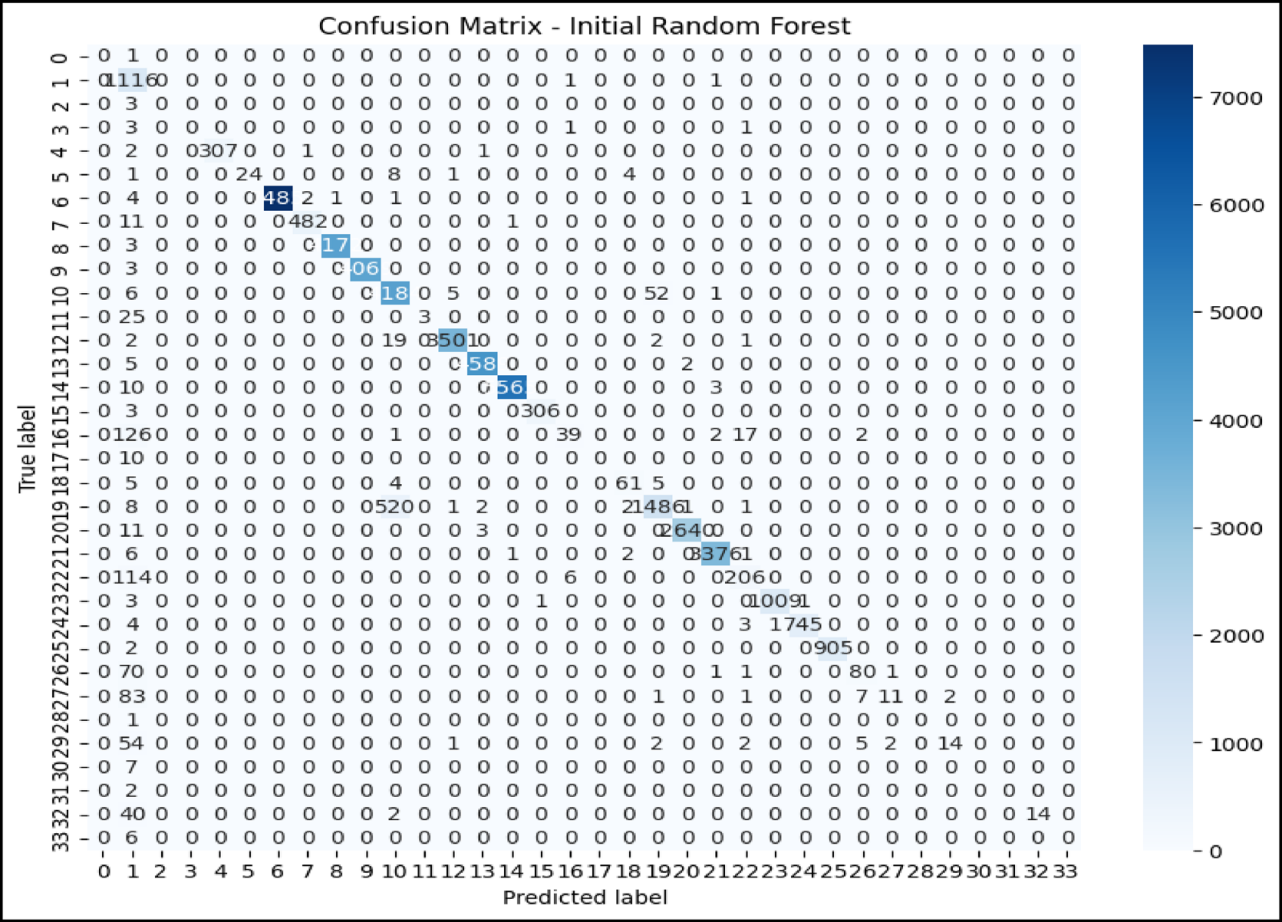
Confusion matrix of initial RF model over CIC-IoT 2023.

**Fig. 14:**
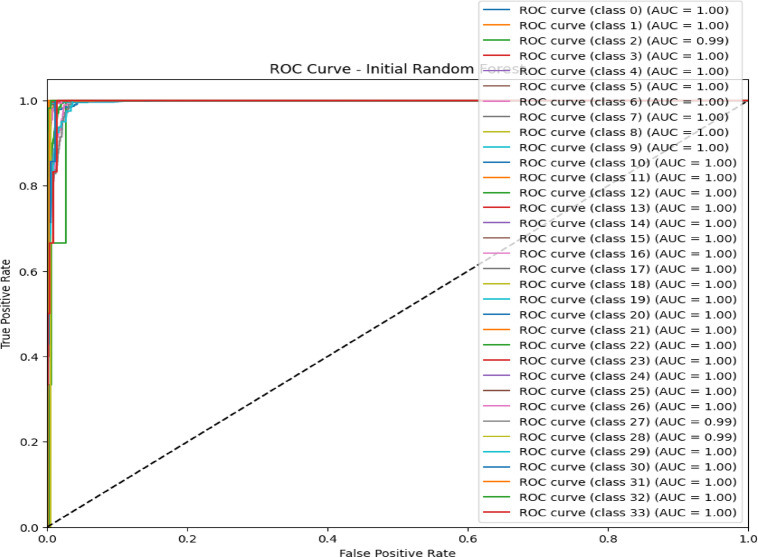
ROC AUC curve of initial RF model over CIC-IoT 2023.

**Fig. 15:**
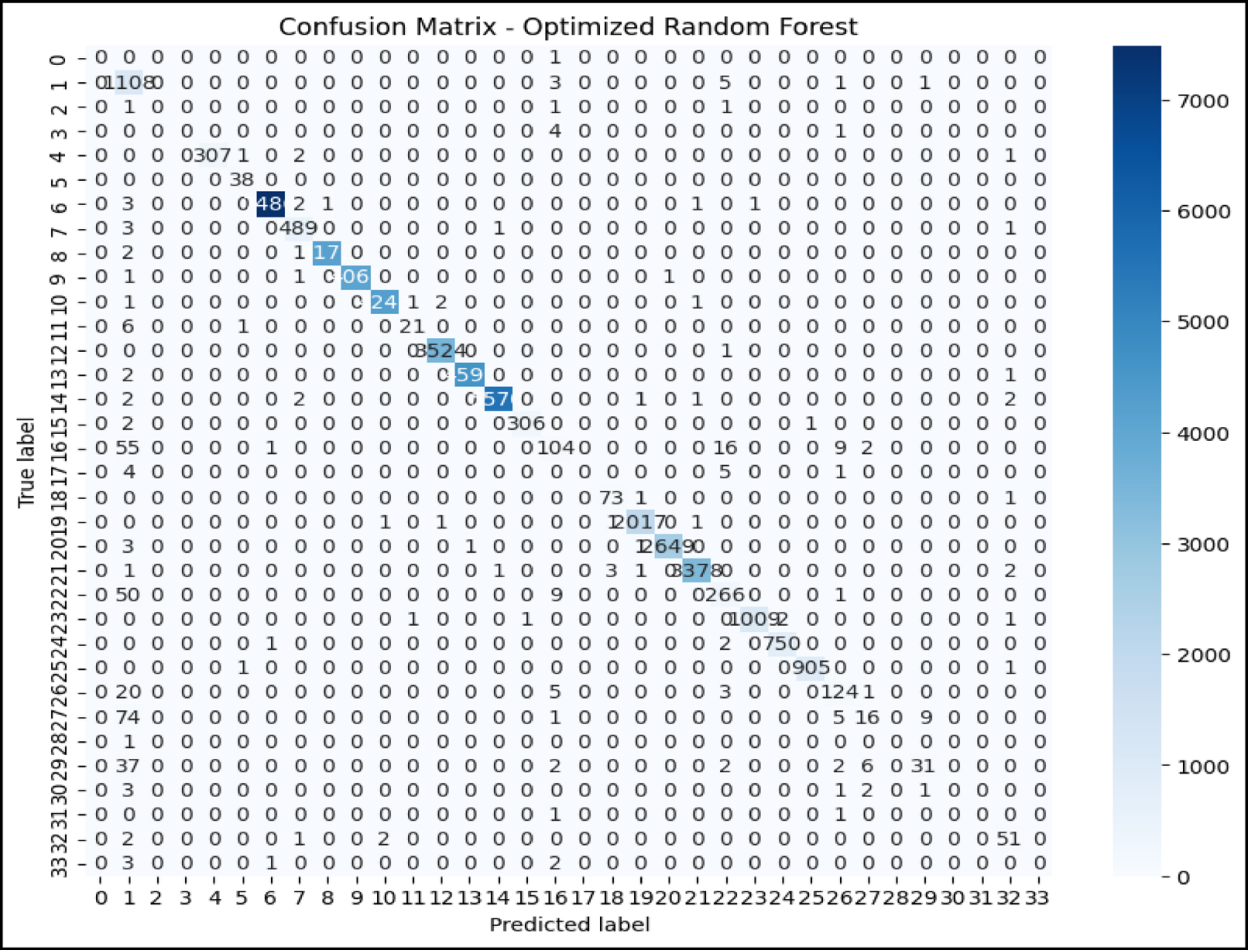
Confusion matrix of optimized RF model over CIC-IoT 2023.

**Fig. 16:**
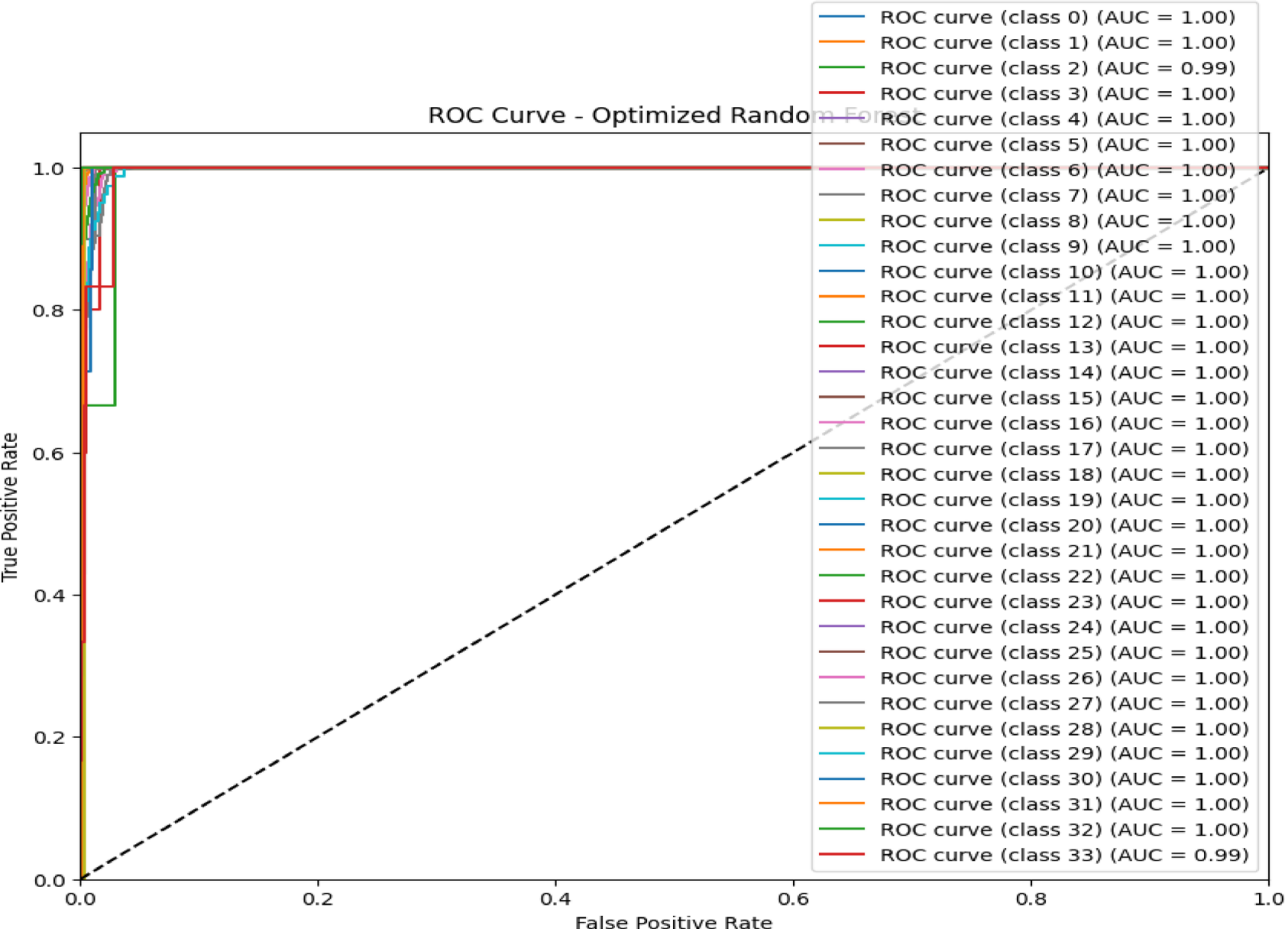
ROC AUC curve of optimized RF model over CIC-IoT 2023.

Figures 13, 14, 15, 16 showcase the evaluation performance of a RF model developed for the CIC-IoT 2023 dataset, comparing performance before optimizations to performance after optimizations. Figure 13 displays the confusion matrix for the RF classifier of the pre-optimization model, showing the number of true positives (TP), false positives (FP), true negatives (TN), and false negatives (FN) to show the classifier’s overall classification performance associated with the pre-optimization model. Figure 14 is the ROC AUC (area under the curve) curve. The AUC curve shows how well the model can discriminate between classes, while AUC reflects how effective the model is overall. Figure 15 displays the confusion matrix for the RF classifier of the post-optimization model, hopefully indicating a more accurate classification performance and better balance of sensitivity and specificity. Figure 16 displays the ROC AUC curve of the post-optimization model, indicating that the model also likely has better discrimination. All four figures allow readers to view the performance of pre- and post-optimization, demonstrating improvements.

### Implications and future directions

The deep analysis of the experiments above reveals several essential insights about the performance of the RF model in the intrusion detection system. It shows good scalability with the performance compared to extensive alternatives for handling large data sizes and exhibiting high value for all metrics. In this case, the optimized model has an outstanding level of reliability of attack detection in terms of its ability to distinguish between two similar patterns of attacks, particularly in such complex Grayhole and TDMA cases, and still maintains low false positives for regular traffic and therefore also provides balanced performance across all types of attacks. From an operational point of view, although the computational complexity does increase during optimization with more trees and a deeper depth, the trade-off is well justified because of the gains obtained in terms of performance with high precision that finally leads to the minimization of false alarms in operations and better-than-chance performance justified by improved Cohen’s Kappa score. Such results show that an optimized model is suitable for real-time network monitoring and automated attack detection systems, thus forming a robust solution for the comprehensive management of WSN security in real-time network monitoring, automated attack detection systems, and policy enforcement in network security.

## Comparison with state-of-the-art techniques

We compared the Tabu Search-optimized Random Forest (TS-RF) model with existing state-of-the-art methods in WSN intrusion detection to demonstrate the effectiveness of the proposed approach. Table 6 shows a comparison of multiple datasets and evaluation metrics.

**Table 6:** Performance comparison of the proposed model against state-of-the-art techniques.

Technique	Dataset	Accuracy	Precision	Recall	F1 Score	Reference
Proposed TS-RF	WSN-DS	0.9967	0.9967	0.9967	0.9967	Our Work
Proposed TS-RF	CICIDS2017	0.9900	0.9900	0.9900	0.9900	Our Work
Proposed TS-RF	CIC-IoT-2023	0.9900	0.9800	0.9900	0.9800	Our Work
DNN	WSN-DS	0.9862	0.9855	0.9862	0.9858	^54^
SVM	WSN-DS	0.9823	0.9830	0.9823	0.9826	^55^
XGBoost	WSN-DS	0.9871	0.9875	0.9871	0.9873	^56^
CNN-LSTM	WSN-DS	0.9758	0.9776	0.9758	0.9767	^57^
Decision Tree	CICIDS2017	0.9873	0.9865	0.9861	0.9863	^58^
LSTM	CICIDS2017	0.9825	0.9811	0.9810	0.9810	^59^
&GNN	CICIDS2017	0.9857	0.9842	0.9835	0.9838	^60^
RF	CIC-IoT-2023	0.9743	0.9725	0.9720	0.9722	^61^
DNN	CIC-IoT-2023	0.9810	0.9755	0.9723	0.9739	^62^
GRU	CIC-IoT-2023	0.9772	0.9755	0.9763	0.9759	^63^

The results clearly show the superiority of our TS-RF model over all published state-of-the-art methods in all datasets. In particular, on the WSN-DS dataset, the TS-RF model outperforms the best competitor (XGBoost^56^) by 1.05%. Regarding network security, there is much more at stake, and any improvement in accuracy, however small, can result in reduced missed detections or false alarms. We see that in the case of the CICIDS2017 dataset, our method has consistently improved approximately 0.43% over the Decision Tree method in^58^. When considering the CIC-IoT-2023 dataset, the TS-RF method had an accuracy improvement of 0.90% compared to the DNN method by Roopak et al.^62^. The superior effectiveness of our approach is a result of the hyperparameter optimization done by Tabu Search, which helps the RF model better fit the complex patterns found in network traffic data without overfitting the training data. This allows the RF to more adequately identify unknowns for imbalanced classes found in intrusion detection datasets; in this regard, it has consistently improved precision, recall, and F1 scores for all attack types. The model also retains the essential computational aspect despite its optimization process. It can be deployed in the most demanding real-time wireless monitor systems (i.e., WSN) lest resources are contaminated, which is commonplace in this field. The slight improvement in detection accuracy without compromising computational performance positions our TS-RF model as an affordable and readily available option for WSN infrastructures to maintain enhanced security.

## Conclusion and future scope

This work successfully demonstrated the efficiency of using Tabu Search optimization on RF intrusion detection for WSNs by making an optimized model that achieved outstanding performance improvements in all the evaluated metrics using the comprehensive dataset provided by WSN-DS, containing 374,661 instances with 19 features. Accuracy was at 99.67% (up from 99.42%), precision at 99.67% (up from 99.43%), recall at 99.67% (up from 99.42%), F1-score at 99.67% (up from 99.42%), Cohen’s Kappa at 0.9808 (up from 0.9667), and ROC AUC at 0.9980 (up from 0.9974).The model notably improved over state-of-the-art detection concerning complex attacks across the different categories of attack in the dataset, including Blackhole, Flooding, Grayhole, and TDMA. The F1 scores of the Blackhole and Grayhole attacks increased from 0.98 to 0.99 and from 0.95 to 0.98, respectively. Further, the proposed model is also tested for the benchmark CCIDS2017 and CIC-IoT-2023 datasets. It was found that the proposed model outperforms each case. In the future, RF can be optimized with the help of some nature-inspired techniques like PSO, Genetic Algorithm, etc.; this approach will include real-time optimization capabilities, emerging patterns through continuous learning mechanisms, and a hybrid optimization technique with multiple metaheuristic algorithms that balance exploration and exploitation well. It will also be interesting to analyze how this model performs in resource-constrained IoT environments. A further possibility of improving practical applicability could be achieved by developing lightweight versions of the optimized model for deployment on sensor nodes with limited computational resourcesand studying the effect of various feature selection methods.

## Data Availability

All data would be available based on the specific requests to corresponding author.
